# Noninvasive Assessment of an Engineered Bioactive Graft in Myocardial Infarction: Impact on Cardiac Function and Scar Healing

**DOI:** 10.5966/sctm.2016-0063

**Published:** 2016-09-02

**Authors:** Carolina Gálvez‐Montón, Ramon Bragós, Carolina Soler‐Botija, Idoia Díaz‐Güemes, Cristina Prat‐Vidal, Verónica Crisóstomo, Francisco M. Sánchez‐Margallo, Aida Llucià‐Valldeperas, Paco Bogónez‐Franco, Isaac Perea‐Gil, Santiago Roura, Antoni Bayes‐Genis

**Affiliations:** ^1^ ICREC (Heart Failure and Cardiac Regeneration) Research Programme, Fundació Institut d'Investigació en Ciències de la Salut Germans Trias i Pujol, Badalona, Barcelona, Spain;; ^2^ Electronic and Biomedical Instrumentation Group, Electronic Engineering Department, Universitat Politècnica de Catalunya, Barcelona, Spain; ^3^ Jesús Usón Minimally Invasive Surgery Centre, Cáceres, Spain; ^4^ Center of Regenerative Medicine in Barcelona, Barcelona, Spain; ^5^ Cardiology Service, Hospital Universitari Germans Trias i Pujol, Badalona, Barcelona, Spain;; ^6^ Department of Medicine, Autonomous University of Barcelona, Barcelona, Spain

**Keywords:** Myocardial infarction, Magnetic resonance imaging, Bioimpedance, Angiogenesis, Progenitor cells

## Abstract

Cardiac tissue engineering, which combines cells and biomaterials, is promising for limiting the sequelae of myocardial infarction (MI). We assessed myocardial function and scar evolution after implanting an engineered bioactive impedance graft (EBIG) in a swine MI model. The EBIG comprises a scaffold of decellularized human pericardium, green fluorescent protein‐labeled porcine adipose tissue‐derived progenitor cells (pATPCs), and a customized‐design electrical impedance spectroscopy (EIS) monitoring system. Cardiac function was evaluated noninvasively by using magnetic resonance imaging (MRI). Scar healing was evaluated by using the EIS system within the implanted graft. Additionally, infarct size, fibrosis, and inflammation were explored by histopathology. Upon sacrifice 1 month after the intervention, MRI detected a significant improvement in left ventricular ejection fraction (7.5% ± 4.9% vs. 1.4% ± 3.7%; *p* = .038) and stroke volume (11.5 ± 5.9 ml vs. 3 ± 4.5 ml; *p* = .019) in EBIG‐treated animals. Noninvasive EIS data analysis showed differences in both impedance magnitude ratio (−0.02 ± 0.04 per day vs. −0.48 ± 0.07 per day; *p* = .002) and phase angle slope (−0.18° ± 0.24° per day vs. −3.52° ± 0.84° per day; *p* = .004) in EBIG compared with control animals. Moreover, in EBIG‐treated animals, the infarct size was 48% smaller (3.4% ± 0.6% vs. 6.5% ± 1%; *p* = .015), less inflammation was found by means of CD25^+^ lymphocytes (0.65 ± 0.12 vs. 1.26 ± 0.2; *p* = .006), and a lower collagen I/III ratio was detected (0.49 ± 0.06 vs. 1.66 ± 0.5; *p* = .019). An EBIG composed of acellular pericardium refilled with pATPCs significantly reduced infarct size and improved cardiac function in a preclinical model of MI. Noninvasive EIS monitoring was useful for tracking differential scar healing in EBIG‐treated animals, which was confirmed by less inflammation and altered collagen deposit. Stem Cells Translational Medicine *2017;6:647–655*

**Significance Statement**

This article describes the development and testing of a new custom‐designed engineered bioimpedance graft (EBIG) to noninvasively monitor myocardial scar healing. The EBIG was made of a scaffold of decellularized human pericardium, green fluorescent protein‐labeled porcine adipose tissue‐derived progenitor cells, and an electrical impedance spectroscopy (EIS) monitoring system. In a noninvasive manner, cardiac function was evaluated by magnetic resonance imaging (MRI) and scar healing by a customized‐design EIS system incorporated within the implanted graft. A significant improvement in MRI left ventricular ejection fraction was detected in EBIG‐treated animals, and histopathology confirmed less inflammation and altered collagen deposit.

## Introduction

Cardiac tissue engineering, which combines cells and biomaterials, is a promising therapy after myocardial infarction (MI) 1. The main goal of this new approach includes both the restoration of damaged tissue and the recovery of cardiac function to limit or prevent adverse ventricular remodeling and end‐stage heart failure. To this end, different biomaterials are being tested (i.e., collagen, alginate, hydrogel, and extracellular matrices) to provide a stable support for cell delivery in close contact with the damaged tissue 2. Microscopic and ultrastructural scaffold topography is key for cellular homing and migration to the target tissue 3, 4. Decellularized tissues offer a natural microenvironment, driving cellular attachment, survival, migration, proliferation, and differentiation 5, 6.

Prat‐Vidal et al. previously reported preliminary data on a novel engineered bioactive impedance graft (EBIG) comprising a scaffold of decellularized human pericardium, green fluorescent protein (GFP)‐labeled porcine adipose tissue‐derived progenitor cells (pATPCs), and an electrical impedance spectroscopy (EIS) monitoring system 7. The functional impact of EBIG in a preclinical model of MI and scar maturation characteristics remains unknown. Accordingly, cardiac function was evaluated in a noninvasive manner by using magnetic resonance imaging (MRI). Additionally, scar healing was evaluated by using a custom‐designed EIS system incorporated within the implanted graft in the acute MI swine model.

## Materials and Methods

### Experimental Design

After a left lateral thoracotomy, an MI was induced via double‐ligation of the first marginal branch of the circumflex artery, 1.5 cm distally from the atrioventricular groove (Prolene 5/0 W‐8556 12‐S, Ethicon, Somerville, NJ, http://www.ethicon.com) 8. After 30 minutes, the EBIG was attached over the infarcted tissue with 0.1–0.2 ml of surgical glue (Glubran 2, CardioLink, Barcelona, Spain, http://www.cardiolink.es). Finally, the animals were allowed to recover and housed for 1 month before sacrifice. In order to ensure that all animals had a similar MI, we analyzed circulating troponin I (cTnI) and creatine kinase in serum samples collected by jugular venipuncture at baseline and 2 hours after MI induction. Cardiac biomarkers were measured in a fluorometric immunoassay analyzer (AQT90 FLEX, Radiometer Medical ApS, Brønshøj, Denmark, http://www.radiometer.com).

**Figure 1:**
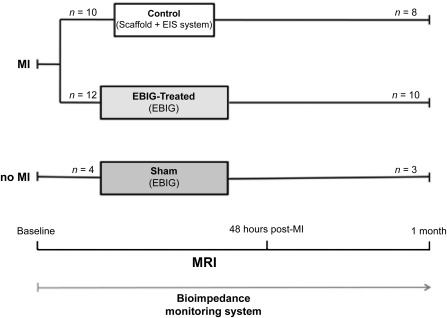
Study design. Abbreviations: EBIG, engineered bioactive impedance graft; EIS, electrical impedance spectroscopy; MI, myocardial infarction; MRI, magnetic resonance imaging; *n*, number of pigs.

Twenty‐six cross‐bred Landrace × large white swine (30.2 ± 3.6 kg) were randomly distributed into three groups (Fig. 1): (a) control arm (*n* = 10), MI induction treated with apposition of a cell‐free pericardial scaffold connected to the EIS system; (b) EBIG‐treated arm (*n* = 12), MI induction treated with the EBIG; and (c) sham arm (*n* = 4), no MI, but the EBIG was implanted on top of healthy myocardium.

All animal studies were approved by the local Animal Experimentation Unit Ethical Committee (no. ES 100370001499) and complied with all guidelines concerning the use of animals in research and teaching as defined by the Guide for the Care and Use of Laboratory Animals (NIH Publication No. 80‐23, revised 1996). Human pericardial samples were obtained after written informed consent from patients undergoing cardiac surgery. The local ethics committee approved this study, and the protocol conformed to the principles outlined in the Declaration of Helsinki.

### Engineered Bioactive Impedance Graft

**Figure 2:**
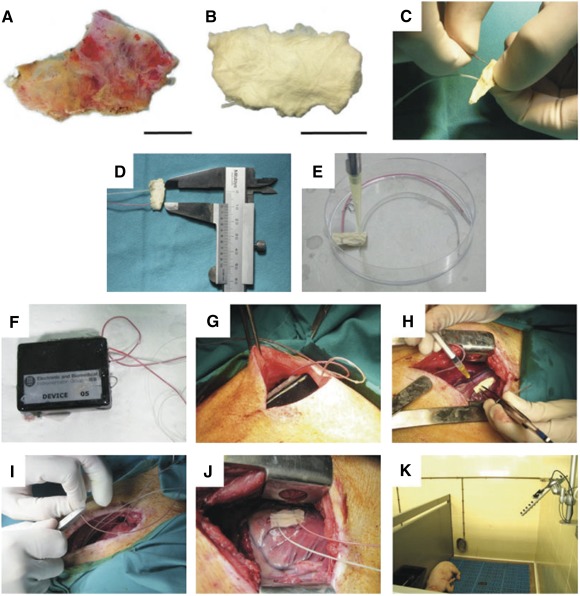
Engineered bioactive impedance graft (EBIG). Photographs show the different steps of EBIG creation and implantation. **(A, B):** Native human **(A)** and decellularized and lyophilized pericardium **(B)**. Scale bars = 1 cm. **(C, D):** Metallic electrodes placement 1 cm spaced. **(E):** Rehydration of the scaffold with or without cells. **(F, G):** Bioimpedance device coated with silicone **(F)** and its implantation in a subcutaneous pocket in the left supraescapular zone **(G)**. **(H, I):** Adhesion of the engineered construct to the myocardial infarction **(H)** and the connection with the electrical impedance spectroscopy system **(I)**. **(J):** Image showing the EBIG implanted in a swine. **(K):** Animal housing under an antenna noninvasively receiving and transmitting the bioimpedance signal.

Human pericardium obtained from patients undergoing cardiac surgery was used as a biological scaffold (Fig. 2A). Pericardial decellularization was performed as previously described 7. Briefly, the tissue was immersed in 1% SDS for 72 hours and then in 1% Triton‐X for an additional 48 hours. Afterward, the membranes were lyophilized with a vacuum pump to extract all fluids, sterilized with γ rays, and stored until use (Fig. 2B). Thirty minutes after MI induction, the metallic electrodes were separately anchored 1 cm apart in the thickness of the membrane (Fig. 2C, 2D). Then, the decellularized pericardium was rehydrated with 175 μl of Puramatrix hydrogel (BD Biosciences, San Jose, CA, http://www.bdbiosciences.com), and in treated animals, the EBIG included 2 × 10^6^ GFP‐pATPCs embedded in 175 μl of 10% sucrose. Finally, to ensure jellification, 350 μl of α‐minimal essential medium was applied (Fig. 2E). Then, the bioimpedance devices, previously coated with biocompatible polydimethylsiloxane silicone and sterilized (Fig. 2F), were located in a subcutaneous pocket in the left supraescapular zone (Fig. 2G). Eighteen EIS systems were implanted (Fig. 2H) (seven animals in each experimental group and four sham animals) and connected to the EIS system (Fig. 2I, 2J) for active monitoring of MI scar evolution. Finally, animals were individually housed with an ad‐hoc‐developed antenna for noninvasive online bioimpedance signal transmission (Fig. 2K).

### Noninvasive Cardiac Function Assessment

Cardiac MRI was performed at 1.5 T (Intera; Philips, Amsterdam, The Netherlands, http://www.usa.philips.com) in all animals by using a four‐channel phased array surface coil (SENSE Body Coil, Philips). Breath‐held, ECG‐gated cine steady‐state precession MRI was acquired (TR/TE 4.1/2.1 ms; flip angle 60°; field of view [FOV] 320 × 320 mm; matrix 160 × 160 pixels; slice thickness 7 mm; bandwidth 1,249.7 Hz per pixel). Delayed enhancement images were acquired after intravenous gadolinium (0.2 ml/kg) using a phase‐sensitive inversion recovery sequence (TR/TE 4.9/1.6 ms; flip angle 15°; inversion time 157 ms; FOV 330 × 330 mm; matrix 224 × 200 pixels; slice thickness 10 mm; bandwidth 282.3 Hz per pixel). Left ventricular ejection fraction (LVEF), cardiac output (CO), stroke volume (SV), left ventricular end‐systolic volume (LVESV), left ventricular end‐diastolic volume (LVEDV), and left ventricular end‐diastolic wall mass (LVEDWM) were measured at baseline, 48 hours after MI, and before sacrifice. Independent blinded investigators carried out MRI data acquisition and analysis.

### Noninvasive Scar Maturation Assessment

Impedance (magnitude and phase angle) was acquired every 5 minutes at 14 frequencies logarithmically spaced between 100 and 200 kHz. System temperature and battery voltage were also acquired. The contents of the EIS systems were downloaded after explantation through the Zigbee radio link controlled by a custom application (LabView, National Instruments, Austin, TX, http://www.ni.com). The impedance measurements were processed with a median filter (*n* = 25, equivalent to 2 hours) to exclude sudden artifacts. Subsequently, a moving average filter (*n* = 150, equivalent to 12.5 hours) was used to smooth the time series by using the zero‐shift double pass filter *filtfilt* (Matlab, MathWorks, Natick, MA, http://www.mathworks.com). In order to correct for long artifacts, the magnitude at the highest frequency (200 kHz) was subtracted from the magnitude at the other frequencies. Two estimators were chosen to display the integrity of the tissue in the monitored area: the slope of the time course of the impedance magnitude ratio between low frequency (LF; 1 kHz) and high frequency (HF; 100 kHz), and the slope of the time course of the phase angle difference between the LF and HF. The rationale for these estimators is presented in Discussion.

### Histopathology Examination

Sacrifices were performed an average of 30.6 ± 3 days after MI with an overdose of anesthesia. After lateral thoracotomy, the hearts were excised. Left ventricle (LV) infarct size was measured by examination of sections obtained 1.5 cm distally to the artery ligation by using the following equation: infarct size (%) = [(LV infarct area)/(LV total area)] × 100. Quantitative morphometric and histological measurements were completed with Image‐Pro Plus software (version 6.2.1; Media Cybernetics, Rockville, MD, http://www.mediacy.com).

On 4‐μm paraffin slices, modified Gallego's and Masson's trichrome and Picrosirius Red staining were performed to analyze both pathological and histological changes and collagen deposition (type I red/yellow and type III green) under a computer‐associated Leica DMI 6000B (Leica, Wetzlar, Germany, http://www2.leicabiosystems.com) microscope with a polarized filter.

Frozen sections of 10 μm were stained by using biotinylated GSLI B4 isolectin (1:25; *Griffonia simplicifolia* lectin I B4, Vector Laboratories, Burlingame, CA, http://vectorlabs.com), smooth muscle actin (SMA; 1:50; Sigma‐Aldrich Química SL, Madrid, Spain, http://www.sigmaaldrich.com), and elastin (1:100; Abcam, Cambridge, MA, http://www.abcam.com) antibodies to quantify vessel area and detect blood vessels within the scaffold. For the inflammatory state study, CD3 (1:100) and CD25 (1:10) (Bio‐Rad, Hercules, CA, http://www.bio‐rad.com) antibodies were applied to determine the presence of lymphocytes and activated lymphocytes, respectively, in the infarct zone. Finally, to study the endothelial and cardiac differentiation of GFP‐pATPCs, and proliferation of cardiomyocytes, anti‐GFP (1:1000: Abcam), c‐Kit (1:50; Bioss, Woburn, MA, http://biossusa.com), MEF‐2 (1:50; MyBiosource, San Diego, CA, http://www.mybiosource.com), cardiac troponin I (1:200; Santa Cruz Biotechnology, Dallas, TX, http://www.scbt.com), NKX2.5 (1:50; Bioss), cardiac troponin T (1:50; Bio‐Rad), SMA (1:50; Sigma‐Aldrich), von Willebrand factor (vWF) (1:100; BD Biosciences, Franklin Lakes, NJ, http://www.bdbiosciences.com), CD31 (1:50; Abcam), and Ki67 (1:100; Santa Cruz Biotechnology) antibodies were used. Alexa Fluor 488‐conjugated streptavidin, Alexa Fluor 488 and 568 (1:500; Thermo Fisher Scientific Life Sciences, Oakwood Village, OH, https://www.thermofisher.com), and Cy2, Cy3, and Cy5 (1:500; Jackson ImmunoResearch Laboratories, West Grove, PA, https://www.jacksonimmuno.com) secondary antibodies were also used. All sections were counterstained with 4′,6‐diamidino‐2‐phenylindole (1:10,000; Sigma‐Aldrich) and analyzed by confocal microscopy (Axio Observer Z1, Zeiss, Oberkochen, Germany, http://www.zeiss.com).

### Statistical Analysis

Data are represented as the mean ± SEM. Statistical analyses were performed by using the Student *t* test, one‐way analysis of variance (ANOVA) with the Tukey's procedure for multiple comparisons, and the Mann‐Whitney test for nonparametric data, using SPSS 19.0.1 (IBM, Armonk, NY, http://www.ibm.com). MRI data were analyzed as repeated measures by using ANOVA with the Greenhouse‐Geisser correction. Bioimpedance data analyses were performed with the paired samples *t* test using SigmaStat software (Systat Software, Inc., San Jose, CA, https://systatsoftware.com). Values of *p* < .05 were considered significant.

## Results

Two animals died during MI induction due to ventricular fibrillation, and three were excluded from the study after postoperative infections. Therefore, 21 animals were included in the experimental protocol in the control (*n* = 8), EBIG‐treated (*n* = 10), and sham (*n* = 3) groups (Fig. 1). Swine were sacrificed at predefined times (*p* = .5 between groups). In all animals, the graft was seen covering the infarct area; absence of deleterious graft‐driven effects was detected in sham animals (supplemental online Fig. 1). Baseline infarct size between EBIG‐treated and control animals was not significantly different as assessed by the necrosis biomarkers cTnI and creatine kinase‐MB (0.39 ± 0.1 vs. 0.32 ± 0.1 μg/l, *p* = .63; and 0.59 ± 0.09 vs. 0.54 ± 0.14 μg/l, *p* = .75, respectively).

### Cardiac Function Assessment

Baseline cardiac function did not differ between the EBIG‐treated and control groups in LVEF (55.8% ± 2.2% vs. 56.5% ± 2.8%; *p* = .85), CO (2.1 ± 0.1 vs. 2.2 ± 0.1 l/min; *p* = .44), SV (24.1 ± 1.6 vs. 25.1 ± 1.3 ml; *p* = .64), LVESV (19.7 ± 2.1 vs. 19.6 ± 1.6 ml; *p* = .96), LVEDV (43.8 ± 3.2 *vs.* 44.7 ± 1.8 ml; *p* = .88), and LVEDWM (42.9 ± 2.2 vs. 44.1 ± 1.6 g; *p* = .68) (supplemental online Table 1).

**Figure 3:**
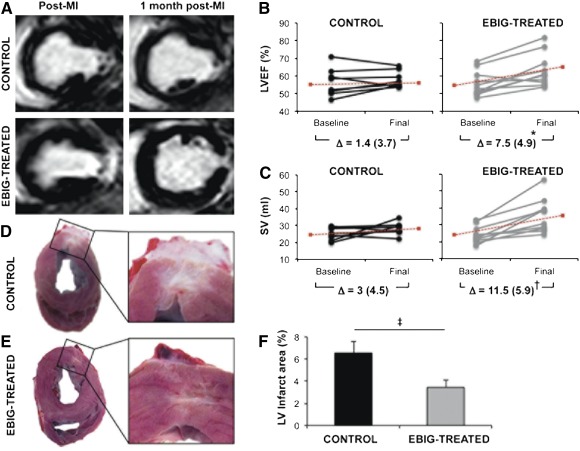
Noninvasive cardiac function and morphometric analysis. **(A):** T1 short‐axis delayed enhancement images from control and EBIG‐treated animal after MI and after 30 days of follow‐up. **(B, C):** LVEF and SV at baseline and 30 days after MI in control and EBIG‐treated animals. Data for individual pigs (dots) and the Δ (±SEM) are shown. ∗, *p* = .038; †, *p* = .019. Dotted red lines indicate the mean evolution over time. **(D, E):** Representative heart sections from control and EBIG‐treated pigs showing the infarcted area of the LV. **(F):** Percentage of the LV infarct area measured in EBIG‐treated pig hearts compared with the control group after 30 days of follow‐up. ‡, *p* = .015. Data represent mean ± SEM. Abbreviations: EBIG, engineered bioactive impedance graft; LV, left ventricle; LVEF, left ventricular ejection fraction; MI, myocardial infarction; SV, stroke volume.

The ANOVA test with Greenhouse‐Geisser post hoc correction showed differences over time in LVEF (7.5% ± 4.9% vs. 1.4% ± 3.7%; *p* = .038) and SV (11.5 ± 5.9 ml vs. 3 ± 4.5 ml; *p* = .019) between EBIG‐treated and control animals, respectively (Fig. 3A–3C). In contrast, no differences were found in CO (*p* = .13), LVESV (*p* = .91), LVEDV (*p* = .07), or LVEDWM (*p* = .56).

To understand the MRI functional benefit in the EBIG‐treated arm, the infarct size and cellular differentiation were assessed. After digital morphometric analysis, LV infarct size was 48% smaller in EBIG‐treated than control animals (3.4% ± 0.6% vs. 6.5% ± 1%, respectively; *p* = .015) (Fig. 3D–3F). Proper adhesion of the implanted graft with subjacent myocardium was observed in all control and EBIG‐treated animals (supplemental online Fig. 2A–2D).

### Cell Proliferation and GFP^+^‐pATPC Endothelial and Cardiac Differentiation

**Figure 4:**
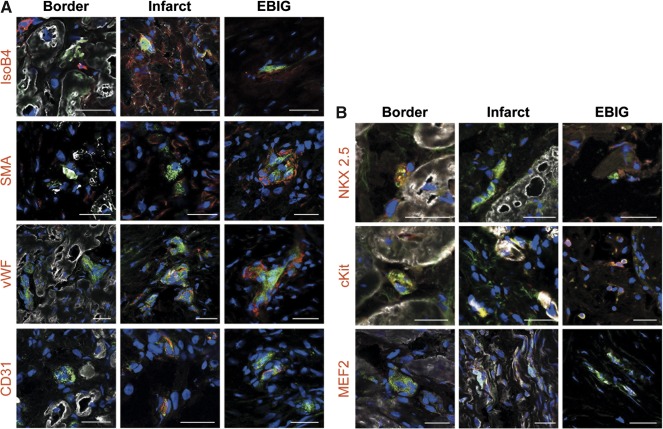
Green fluorescent protein‐adipose tissue‐derived progenitor cell (GFP‐ATPC) endothelial and cardiac differentiation. **(A):** Immunohistochemistry images from border, infarct, and EBIG zones of treated animals showing positive GFP‐ATPCs (green) and IsoB4, SMA, vWF, and CD31 (red) endothelial antibodies. **(B):** Representative images from border, infarct, and EBIG zones of treated animals showing positive GFP‐ATPCs (green) and NKX2.5, cKit, and circulating troponin I (white), and MEF2 (red) and cardiac troponin T (white), and cardiac markers. Nuclei are counterstained with 4′,6‐diamidino‐2‐phenylindole (blue). Scale bars = 50 μm. Abbreviations: EBIG, engineered bioactive impedance graft; IsoB4, GSLI B4 Isolectin; SMA, smooth muscle actin; vWF, von Willebrand factor.

As supplemental online Figure 3 depicts, there were cardiomyocytes expressing the cell proliferative marker Ki67 in the border zone of treated animals. However, Ki67‐positive cells were not detected in the infarct core. Additionally, upon sacrifice, grafts from EBIG‐treated and control animals showed vessel formation, some of which were positive for SMA, which is indicative of the existence of a vascular media layer (supplemental online Fig. 2E, 2F). Moreover, GFP^+^‐pATPCs de novo expressed the endothelial markers IsoB4, SMA, vWF, and CD31^+^ within the EBIG and at the infarct zone, suggesting graft‐driven neovascularization (Fig. 4A). Relative to cardiac differentiation analysis, we found that both GFP^+^‐pATPCs within the graft and those that migrated to the infarct area were positive for NKX2.5, cKit, MEF2, cTnI, and cTnT. (Fig. 4B).

### Scar Evolution Monitoring

Out of the 18 systems implanted (7 per experimental group and 4 in the sham group), 11 provided useful data (4 controls, 4 EBIG‐treated, and 3 sham). Four of the remaining EIS systems were in deceased or excluded animals, and the remaining three stopped measuring after 1–3 days for unknown reasons.

**Figure 5:**
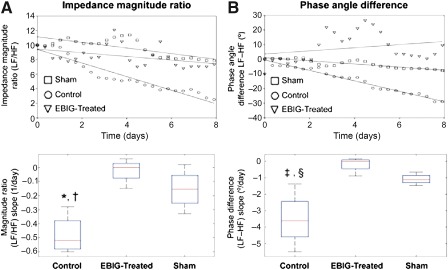
Electrical impedance spectroscopy‐derived tissue‐state evolution estimators. Time course of the impedance magnitude ratio **(A)** and phase angle difference **(B)** in the control, EBIG‐treated, and sham groups is shown. The curves show 1 of every 100 measurement points for sake of clarity and represent the average curve for each group (control, circles, average of *n* = 4; EBIG‐treated, triangles, average of *n* = 4; sham, squares, average of *n* = 3). The three curves have been normalized to have the same origin to make the comparison easier. The dotted regions around the regression lines represent the 95% confidence interval for the regression. Both the magnitude ratio and phase angle difference show a clearly decreasing slope for the control group, whereas they remain almost constant for the EBIG‐treated and sham groups. These results are consistent with the transition to scar tissue for the control group and the existence of normal‐like tissue in the EBIG‐treated and sham groups. **(A):** At the bottom is the box plot of the magnitude ratio slope estimator. ∗, *p* = .002; †, *p* = .013. **(B):** At the bottom appears the box plot of the phase angle difference slope estimator, which also displayed a significant difference between controls and the other two groups. ‡, *p* = .004; §, *p* = .03. The means and confidence intervals can be found in Results. Abbreviations: EBIG, engineered bioactive impedance graft; HF, high frequency; LF, low frequency.

Within 8 days, significant differences were observed between EBIG‐treated and control animals. Figure 5 shows the time course of the impedance magnitude ratio (Fig. 5A) and phase angle difference (Fig. 5B) in the three groups. The magnitude ratio slope estimator was −0.48 ± 0.07 per day for controls and −0.02 ± 0.04 per day for EBIG‐treated (*p* = .002). There was also a significant difference between the control and sham groups (−0.15 ± 0.07 per day; *p* = .013), whereas no differences were found between the EBIG‐treated and sham groups (*p* = .39).

Regarding the phase angle difference slope estimator, the results were −3.52° ± 0.84° per day for controls and −0.18° ± 0.24° per day for EBIG‐treated (*p* = .004). There was also a significant difference between the control and sham groups (−1.08° ± 0.17° per day; *p* = .03). The slopes were calculated as the first‐order coefficient of the linear regression for every magnitude ratio and phase difference time courses without any normalization. Figure 5 shows box plots of the magnitude ratio slope estimator (Fig. 5A) and the phase angle difference slope estimator (Fig. 5B).

**Figure 6:**
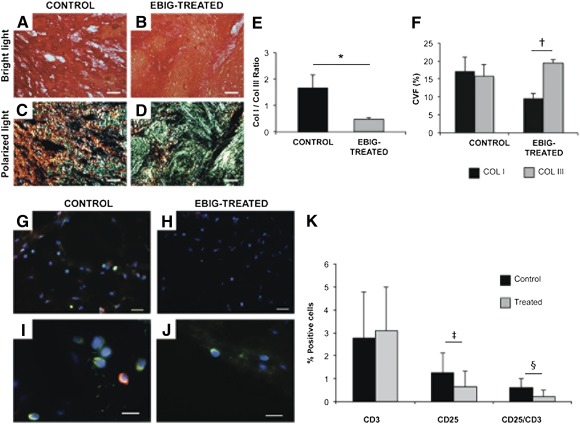
Myocardial fibrosis and inflammation. **(A, B):** Sirius red‐stained images exhibiting collagen (red) and healthy muscle (yellow) in the infarct core from control and EBIG‐treated animals. **(C, D):** Polarized light microscopy images show fibrils of collagen I (red/yellow) and collagen III (green) in the same sections. Scale bars = 50 μm. **(E):** Collagen I/III ratio measured on polarized light images. ∗, *p* = .019. **(F):** Percentage of collagen I and III measured on polarized light images. †, *p* < .001. **(G–J):** Representative images of CD3 (green) and CD25 (red) at ×400 **(G, H)** and ×630 **(I, J)** in the infarct core from control and EBIG‐treated groups. Scale bars = 50 μm **(G, H)** and 20 μm **(I, J)**. **(K):** Percentage of CD3‐ and CD25‐positive cells and CD25/CD3 ratio. ‡, *p* = .001; §, *p* < .001. Data represent the mean ± SEM measurement. Abbreviations: ColI, collagen I; Col III, collagen III; CVF, collagen volume fraction; EBIG, engineered bioactive impedance graft.

To correlate the EIS findings with tissue differences between groups, collagen content and inflammation were examined by using histopathological techniques. Collagen content was measured after Picrosirius red staining. Upon sacrifice, collagen I (9.6% ± 1.4% vs. 17.2% ± 3.9%; *p* = .1), collagen III (19.5% ± 1.0% vs. 15.8% ± 3.3%; *p* = .31), collagen volume fraction (29.1% ± 1.9% vs. 32.9% ± 4.6%; *p* = .46), and the collagen I/III ratio (0.5 ± 0.1 vs. 1.7 ± 0.5; *p* = .019) were analyzed in the infarct cores of EBIG‐treated compared with control animals, respectively (Fig. 6A–6E). The inverse collagen I/III ratio in EBIG‐treated animals was also confirmed when relative content of collagen I and III were comparatively assessed in control (17.2% ± 3.9% vs. 15.8% ± 3.3%, respectively; *p* = .79) and EBIG‐treated (9.6% ± 1.4% vs. 19.5% ± 1.0%, respectively; *p* < .001) animals (Fig. 6F).

### Vascular Density and Inflammation Analyses

After Isolectin B4 staining, no differences in the vascularized area between treated and control MI groups in remote (1.66% ± 0.24% vs. 1.3%8 ± 0.21%, respectively; *p* = .41), infarct (0.9% ± 0.27% vs. 0.87% ± 0.48%, respectively; *p* = .96), and border (1.08% ± 0.16% vs. 0.79% ± 0.18%, respectively; *p* = .32) zones were found (data not shown). After 30 days, in all implanted bioprostheses some SMA‐positive vessels were detected (supplemental online Fig. 2E, 2F). Analysis of inflammation at the infarct core showed no differences in CD3^+^ content (*p* = .49) between groups; however, CD25^+^content (0.65 ± 0.12 vs. 1.26 ± 0.20; *p* = .001) and the CD25^+^/CD3^+^ ratio (0.23 ± 0.04 vs. 0.61 ± 0.06; *p* < .001) were lower in EBIG‐treated animals (Fig. 6G–6K).

## Discussion

In the present study, we focused on a noninvasive assessment of cardiac function and scar healing using cardiac MRI and EIS to observe changes induced by a novel EBIG applied in a preclinical model of MI in swine. Two main conclusions emerge from our results: First, the increase in cardiac contractility (assessed by LVEF) in the EBIG‐treated group was more than fivefold higher than that of controls; second, the EBIG positively impacted the healing process, providing impedance values similar to those of sham‐operated animals, and altered scar collagen content.

Indeed, the remarkable cardiac function benefits with the EBIG in terms of LVEF and SV improvement by MRI analysis are in agreement with the 48% reduction in infarct size in EBIG‐treated animals. Similar, but lower‐magnitude, benefits were previously described when adipose‐derived progenitor cells were implanted using different routes of administration (intramyocardial injection 9, 10, 11, intracoronary infusion 11, or peripheral intravenous delivery 12) in murine and swine MI models. Adipose‐derived progenitor cells tested in cardiac regeneration therapies may be of subcutaneous 10, 11, 12 or cardiac origin 9, 13. Bayes‐Genis et al. previously demonstrated that cardiac adipose‐derived progenitors were more committed toward a cardiac‐like phenotype than those of subcutaneous origin 9. Moreover, when seeded into the scaffold, these cells showed repopulation and viability levels suitable for in vivo implantation 7. In this study, upon sacrifice, we were able to find GFP^+^ cells within both the EBIG and in underlying myocardium de novo expressing cTnI and NKX2.5. cTnI plays a key role in mature sarcomere organization, and NKX2.5 is central in activating the cascade of cardiomyogenic genes. Thus, it is likely that pATPCs are partly responsible for the beneficial effects of the EBIG because many studies have reported that ATPCs secrete factors such as adipokines, growth factors, and proteins from the extracellular matrix that exert an important role in cardiac regeneration 14. Evidence is also accumulating showing that adipose‐derived hormones can offer cardioprotective effects, including attenuation of cardiomyocyte apoptosis and reduction of infarct size 8. Finally, neovascularization of the graft and underlying myocardium has also been suggested as a putative mechanism to limit infarct size 6. In line with this, despite no vessel density differences in infarct core, within the graft, the scar, and the remote myocardium we found vessels positive for GFP, IsoB4, SMA, vWF, and CD31, but not in remote myocardium. In point of fact, GFP^+^ pAPTCs within the scaffold also expressed SMA. Regarding cardiomyocyte proliferation, and in agreement with previous studies 15, the limited number of newly formed cardiomyocytes identified in EBIG‐treated animals may only have a low contribution to the significant improvement observed.

Interestingly, to noninvasively monitor online myocardial scar evolution over 30 days, the grafts of sham, control, and EBIG‐treated animals were connected to a customized EIS. Previous measurements of in vitro and in vivo myocardium in both normal and healed infarction states have been reported, displaying a frequency‐dependent higher impedance for normal tissue and a resistive behavior for scar tissue 16, 17. Consequently, a gradual evolution between both states, with time‐decreasing impedance at low frequencies, could be expected in the transition to healed scar tissue in control animals, whereas impedances at all frequencies should remain constant in EBIG‐treated animals. Our initial aim was to fit all measurements to the Cole model for impedance and to study the time course of the model parameters to obtain information about the structural changes undergone by the tissue. The difficulty in performing suitable calibration because of differences in electrode impedances and in capacitive coupling between the cables and the animal's body forced us to abandon this goal and to define estimators based on low‐ and high‐frequency measurements. The variability between subjects, as well as possible differences in the cell constant defined by interelectrode distance, made it necessary to define relative estimators like the magnitude ratio and phase angle difference between low‐ and high‐frequency measurements. Finally, the slope of the time course of both the magnitude ratio and phase difference showed significant differences between the control and the EBIG‐treated/sham animals. According to EIS results, the main healing changes occurred before the 8th day after the infarct, as shown by ample histopathology evidence 18. Both the magnitude ratio and phase angle difference showed a clearly decreasing slope for the control group, whereas it remained almost constant for the EBIG‐treated and sham groups. These results are consistent with the transition to scar tissue in the control group and the preservation of myocardial tissue (at least from an impedance perspective) in EBIG‐treated animals. In accordance, analysis of myocardial fibrosis on the infarct core showed a lower collagen I/III ratio in EBIG‐treated animals due to less collagen I deposition and higher collagen III, which is eventually replaced by healthy tissue.

Because inflammation is also a key step in myocardial healing after MI injury, we explored the impact of EBIG as an inflammatory modulator. Indeed, fewer activated lymphocytes accumulated in EBIG‐treated animals, a phenomenon most likely caused by the adipose progenitors contained within the graft. Perea‐Gil et al. have reported the immunomodulatory potential of cardiac adipose tissue progenitors abrogating T‐cell alloproliferation in vitro 19. A key event in graft rejection is the activation and proliferation of the recipient's lymphocytes, particularly T cells. Inhibition of T‐cell activation (CD25^+^) is observed in patients under immunosuppressive treatment 20.

## Conclusion

An advanced EBIG composed of acellular human pericardium refilled with pATPCs significantly reduced infarct size and improved cardiac function in a preclinical model of MI. In this context, the integration of a sophisticated noninvasive EIS monitoring system was useful to analyze the evolution of myocardial healing in EBIG‐treated animals. Collectively, from an EIS perspective, myocardial tissue was preserved in EBIG‐treated animals despite MI induction, which was confirmed by histopathological measurements of less inflammation and altered collagen I/III ratio.

## Author Contributions

C.G.‐M.: conception and design, animal experimentation, collection and/or assembly of data, data analysis and interpretation, manuscript writing; R.B.: collection and/or assembly of data, data analysis and interpretation, bioimpedance device creation, final approval of manuscript; C.S.‐B. and I.D.‐G.: collection and/or assembly of data, data analysis and interpretation, animal experimentation; C.P.‐V., V.C., and A.L.‐V.: collection and/or assembly of data, data analysis and interpretation; F.M.S.‐M.: provision of study material, final approval of manuscript; P.B.‐F.: collection and/or assembly of data, data analysis and interpretation, bioimpedance device creation; I.P.‐G.: collection and/or assembly of data; S.R.: manuscript writing, final approval of manuscript; A.B.‐G.: conception and design, manuscript writing, final approval of manuscript.

## Disclosure of Potential Conflicts of Interest

The authors indicated no potential conflicts of interest.

## Supporting information

Supporting Information

## References

[1] Vunjak‐Novakovic G , Tandon N , Godier A et al. Challenges in cardiac tissue engineering. Tissue Eng Part B Rev 2010;16:169–187. 19698068 10.1089/ten.teb.2009.0352 PMC2946883

[2] Gálvez‐Montón C , Prat‐Vidal C , Roura S et al. Update: Innovation in cardiology (IV). Cardiac tissue engineering and the bioartificial heart. Rev Esp Cardiol (Engl Ed) 2013;66:391–399. 24775822 10.1016/j.rec.2012.11.012

[3] Moroni F , Mirabella T . Decellularized matrices for cardiovascular tissue engineering. Am J Stem Cells 2014;3:1–20. 24660110 PMC3960753

[4] Badylak SF , Freytes DO , Gilbert TW . Extracellular matrix as a biological scaffold material: Structure and function. Acta Biomater 2009;5:1–13. 18938117 10.1016/j.actbio.2008.09.013

[5] Midwood KS , Williams LV , Schwarzbauer JE . Tissue repair and the dynamics of the extracellular matrix. Int J Biochem Cell Biol 2004;36:1031–1037. 15094118 10.1016/j.biocel.2003.12.003

[6] Gálvez‐Montón C , Fernandez‐Figueras MT , Martí M et al. Neoinnervation and neovascularization of acellular pericardial‐derived scaffolds in myocardial infarcts. Stem Cell Res Ther 2015;6:108. 26205795 10.1186/s13287-015-0101-6 PMC4529715

[7] Prat‐Vidal C , Gálvez‐Montón C , Puig‐Sanvicens V et al. Online monitoring of myocardial bioprosthesis for cardiac repair. Int J Cardiol 2014;174:654–661. 24820760 10.1016/j.ijcard.2014.04.181

[8] Gálvez‐Montón C , Prat‐Vidal C , Roura S et al. Transposition of a pericardial‐derived vascular adipose flap for myocardial salvage after infarct. Cardiovasc Res 2011;91:659–667. 21576133 10.1093/cvr/cvr136

[9] Bayes‐Genis A , Soler‐Botija C , Farré J et al. Human progenitor cells derived from cardiac adipose tissue ameliorate myocardial infarction in rodents. J Mol Cell Cardiol 2010;49:771–780. 20713059 10.1016/j.yjmcc.2010.08.010

[10] Soler‐Botija C , Bagó JR , Llucià‐Valldeperas A et al. Engineered 3D bioimplants using elastomeric scaffold, self‐assembling peptide hydrogel, and adipose tissue‐derived progenitor cells for cardiac regeneration. Am J Transl Res 2014;6:291–301. 24936221 PMC4058310

[11] Gautam M , Fujita D , Kimura K et al. Transplantation of adipose tissue‐derived stem cells improves cardiac contractile function and electrical stability in a rat myocardial infarction model. J Mol Cell Cardiol 2015;81:139–149. 25724725 10.1016/j.yjmcc.2015.02.012

[12] Rigol M , Solanes N , Farré J et al. Effects of adipose tissue‐derived stem cell therapy after myocardial infarction: Impact of the route of administration. J Card Fail 2010;16:357–366. 20350704 10.1016/j.cardfail.2009.12.006

[13] Jun Hong S , Rogers PI , Kihlken J et al. Intravenous xenogeneic transplantation of human adipose‐derived stem cells improves left ventricular function and microvascular integrity in swine myocardial infarction model. Catheter Cardiovasc Interv 2015;86:E38–E48. 24905889 10.1002/ccd.25566 PMC5896329

[14] Kokai LE , Marra K , Rubin JP . Adipose stem cells: Biology and clinical applications for tissue repair and regeneration. Transl Res 2014;163:399–408. 24361334 10.1016/j.trsl.2013.11.009

[15] Anversa P , Leri A . Innate regeneration in the aging heart: Healing from within. Mayo Clin Proc 2013;88:871–883. 23910414 10.1016/j.mayocp.2013.04.001 PMC3936323

[16] Schwartzman D , Chang I , Michele JJ et al. Electrical impedance properties of normal and chronically infarcted left ventricular myocardium. J Interv Card Electrophysiol 1999;3:213–224. 10490477 10.1023/a:1009887306055

[17] Warren M , Bragós R , Casas O et al. Percutaneous electrocatheter technique for on‐line detection of healed transmural myocardial infarction. Pacing Clin Electrophysiol 2000;23:1283–1287. 10962753 10.1111/j.1540-8159.2000.tb00945.x

[18] Mitchell RS , Kumar V , Abbas AK et al. Robbins Basic Pathology. 8th ed. Philadelphia, PA: Saunders, 2009.

[19] Perea‐Gil I , Monguió‐Tortajada M , Gálvez‐Montón C et al. Preclinical evaluation of the immunomodulatory properties of cardiac adipose tissue progenitor cells using umbilical cord blood mesenchymal stem cells: A direct comparative study. Biomed Res Int 2015;2015:439808. 25861626 10.1155/2015/439808 PMC4377370

[20] Shipkova M , Wieland E . Surface markers of lymphocyte activation and markers of cell proliferation. Clin Chim Acta 2012;413:1338–1349. 22120733 10.1016/j.cca.2011.11.006

