# Supplementary material for: Noninvasive Assessment of an Engineered Bioactive Graft in Myocardial Infarction: Impact on Cardiac Function and Scar Healing
Source: Stem Cells Transl Med. 2016 Sep 2;6(2):647–55. doi: 10.5966/sctm.2016-0063 (PMC5442807; doi:10.5966/sctm.2016-0063)
Supplement: Supplementary file 1 — Supporting Information [file SCT3-6-647-s001.pdf]

Supplemental Information - Gálvez-Montón et al.

Table S1. MRI data analysis. LVEF=Left Ventricular Ejection Fraction; CO=Cardiac Output; SV=Stroke Volume; EDV=End Diastolic Volume; ESV=End Systolic Volume; EDWM=End Diastolic Wall Mass.

	**Baseline**		**48 hours post-MI**		**1 month post-MI**	
	**Control**	**EBIG-Treated**	**Control**	**EBIG-Treated**	**Control**	**EBIG-Treated**
**LVEF**	56.5±2.8	55.8±2.3	52.8±1.6	53.2±1.9	57.9±1.7	63.3±2.9
**CO**	2.2±0.1	2.1±0.1	2±0.1	1.8±0.1	2.4±0.1	2.6±0.2
**SV**	25.1±1.3	24.1±1.6	24.1±1.3	24.6±1.5	28.2±1.3	35.6±3.1
**EDV**	44.7±1.8	43.8±3.2	45.6±1.8	46.7±3.2	48.9±2.5	56.3±4.5
**ESV**	19.6±1.6	19.7±2.1	21.5±1	22.2±2.1	20.7±1.6	20.7±2.3
**EDWM**	44.1±1.6	42.9±2.2	50.4±2.2	50.4±1.2	53±1	53.2±1.6

FIGURE LEGENDS

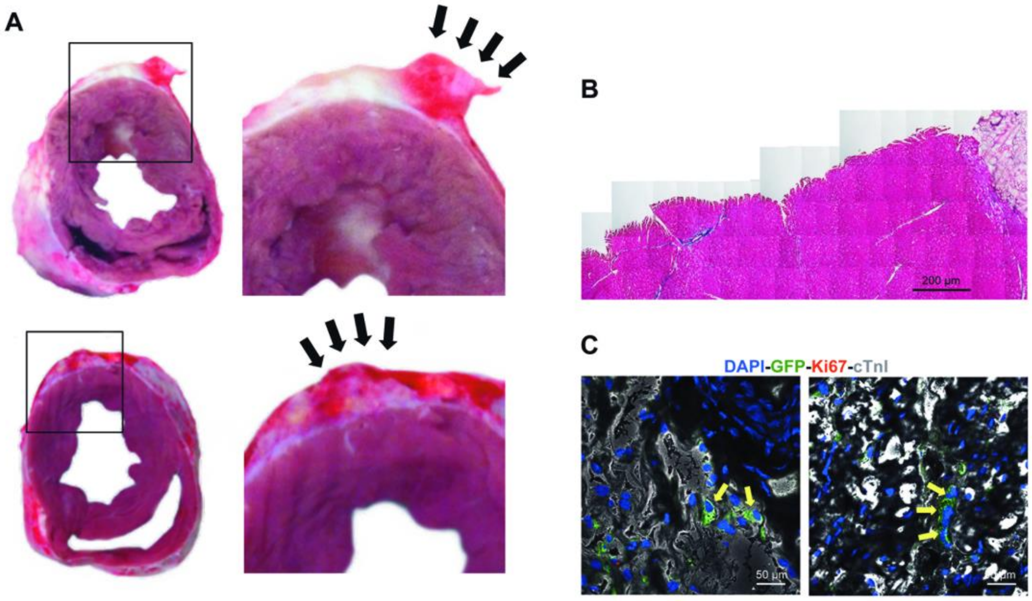

Figure S1. Myocardial analyses of sham animals. (A) Representative heart sections from two sham animals showing the myocardium and the EBIG after 30 days of follow-up. At the left the corresponding zoom images exhibiting in detail the EBIG (arrows). (**B**) Masson's trichrome staining composition screening demonstrated the EBIG adhesion and the healthy adjacent myocardium of a sham animal (scale bar = 200 µm). (**C**) Immunostaining against GFP (green), Ki67 (red), and cTnI (white) antibodies showing the migration of GFP-pATPCs into the healthy myocardium 30 days post-EBIG implantation. Nuclei are counterstained with DAPI (blue) (scale bars = 50 µm).

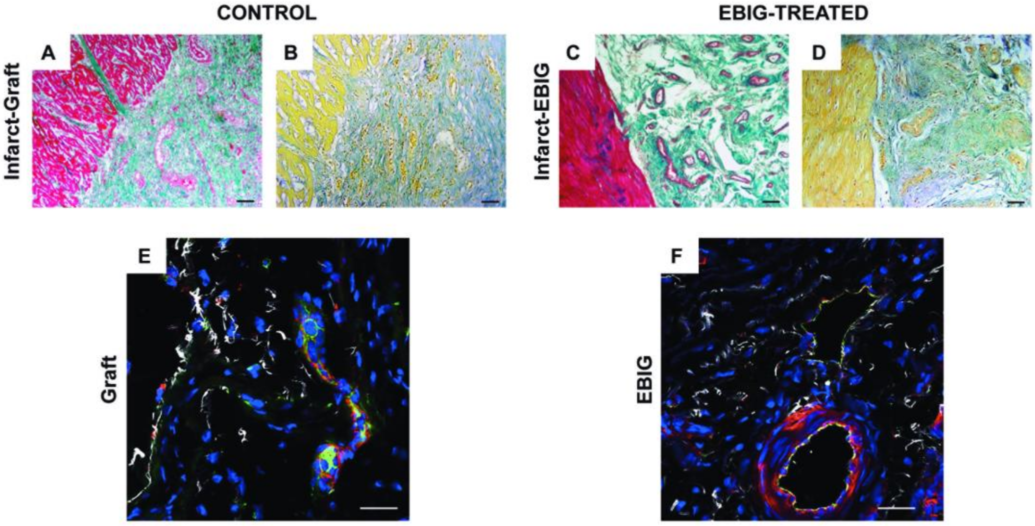

Figure S2. Histological analysis and newly formed vessels in the graft. (**A-D**). Histological analysis after light green Masson's and Gallego's modified trichrome staining showing the myocardium-scaffold interface. (**F and G**) Images of scaffolds labeled with elastin (white) after 30 days of implantation of an animal in the control group (**E**) and a treated (**F**) animal with newlyformed vessels positive for isolectin B4 (green) and SMA (red). Nuclei are counterstained with DAPI (blue) (scale bar = 50 µm).

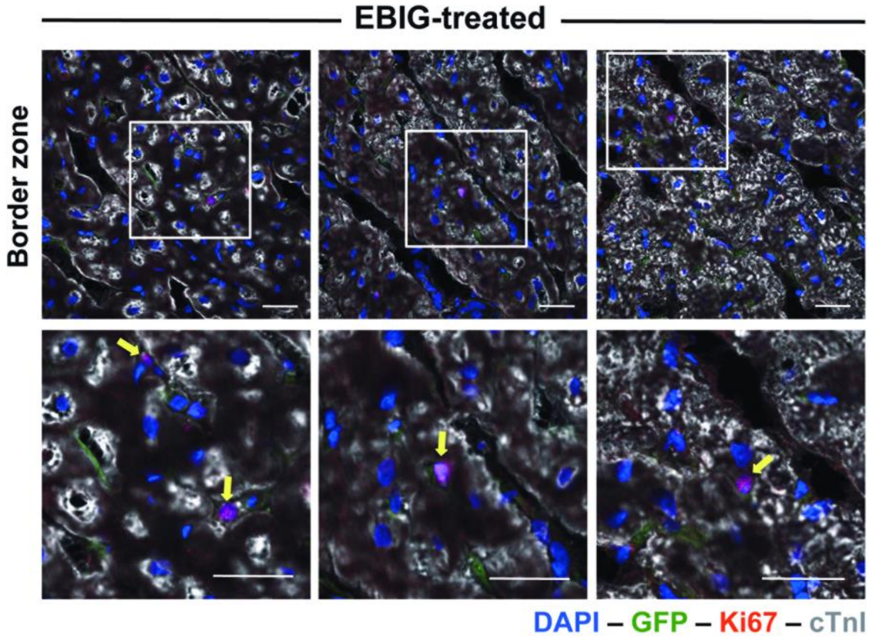

Figure S3. Cardiomyocyte proliferation. Immunohistochemistry images from border zones of EBIG-treated animals against GFP (green), Ki67 (red), and cTnI (white) antibodies showing host proliferating cardiomyocytes (yellow arrows). Nuclei are counterstained with DAPI (blue) (scale bars=50 µm).
